# Biomarkers for early diagnosis of Alzheimer disease: ‘ALZheimer ASsociated gene’– a new blood biomarker?

**DOI:** 10.1111/j.1582-4934.2008.00313.x

**Published:** 2008-03-19

**Authors:** Kurt A Jellinger, Bernd Janetzky, Johannes Attems, Elisabeth Kienzl

**Affiliations:** a Institute of Clinical NeurobiologyVienna, Austria; b Neurological Clinic, Technical UniversityDresden, Germany; c Department of Pathology, OWS HospitalVienna, Austria; d TU Biomed-Neuroscience, Technical University of ViennaAustria

**Keywords:** Alzheimer disease, dementia, ALZAS protein, serum ELISA test, biomarker

## Abstract

Simple, non-invasive tests for an early detection of degenerative dementia by use of biomarkers are urgently required. However, up to the present, no validated extracerebral diagnostic markers (plasma/serum, platelets, urine, connective tissue) for the early diagnosis of Alzheimer disease (AD) are available. In disease stages with evident cognitive disturbances, the clinical diagnosis of probable AD is made with around 90% accuracy using modern clinical, neuropsychological and imaging methods. Diagnostic sensitivity and specificity even in early disease stages are improved by CSF markers, in particular combined tau and amyloid β peptides (Aβ) and plasma markers *(e.g.* Aβ-42/Aβ-40 ratio). Recently, a novel gene/protein – ALZAS (ALZheimer ASsociated protein) – with a 79 amino acid sequence, containing the amyloid β-42 fragment (Aβ-42), the amyloid precursor protein (APP) transmembrane signal and a 12 amino acid C-terminal, not present in any other known APP alleles, has been discovered on chromosome 21 within the APP region. Reverse transcriptase-PCR revealed the expression of the transcript of this protein in the cortex and hippocampal regions as well as in lymphocytes of human AD patients. The expression of ALZAS is mirrored by a specific autoimmune response in AD patients, directed against the ct-12 end of the ALZAS-peptide but not against the Aβ-sequence. ELISA studies of plasma dectected highest titres of ALZAS in patients with mild cognitive impairment (presymptomatic AD), but only moderately increased titres in autopsy-confirmed AD, whereas low or undetectable ct-12 titres were found in cognitively intact age-matched subjects and young controls. The antigen, ALZAS protein, was detected in plasma in later clinical stages of AD. It is suggested that ALZAS represents an indicator in a dynamic equilibrium between both peripheral and brain degenerative changes in AD and may become a useful ‘non-invasive’ diagnostic marker *via* a simple blood test.

- Introduction
- Pathogenesis of AD
- Alzheimer pathology and diagnosis
- Biomarkers of AD
  - CSF biomarkers
  - Plasma biomarkers
  - Platelet biomarkers
  - Structural biomarkers
- ALZheimer ASsociated protein (ALZAS)
  - ALZAS immunohistochemistry
  - ALZAS-mRNA studies
  - ALZAS as a future biomarker?
- Conclusions

## Introduction

Alzheimer disease (AD) is the most common cause of dementia in the elderly, accounting for 65–70% of all cases [1]. The lifetime risk for AD between age 65 and 100 is 33% for men and 45% for women with an annual increase of 1–2% in the seventh decade to almost 60% in the 10^th^ decade with doubling every 5 years. AD is very common and thus is a major public health problem. As the world's population ages, the number of people with AD is expected to increase dramatically from approximately 24 million people in 2001 up to 81 million by 2040 [2]. The total worldwide yearly costs for the treatment and care of demented patients are estimated around 250 billion US dollars [3]. Dementia, after cardiovascular disease and malignancies, is the third frequent cause of death.

**Fig. 1:**
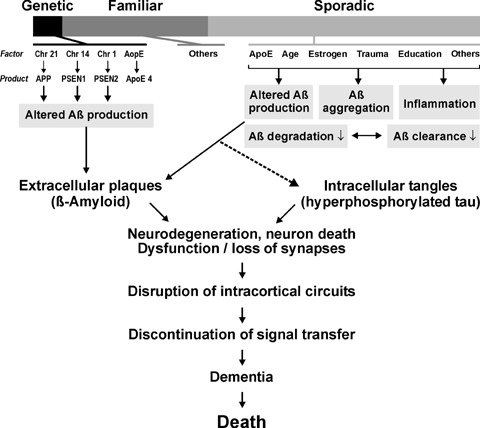
Major pathogenic factors causing Alzheimer disease.

From a genetic viewpoint, AD is a heterogeneous disorder with both (rare) familial and (frequent) sporadic forms. Familial AD (FAD), representing less than 2% of the total, is an autosomal dominant disorder with onset before age 65 years, caused by mutations of the APP gene on chromosome 21 [4], and, less frequently, mutations in the highly homologous presenilin 1 (PSEN1) on chromosome 14, and presenilin 2 (PSEN2) on chromosome 1 [5–7]. While these latter forms are rare with a prevalence below 0.1%[8], a number of genes, *e.g.* the apolipoprotein E (APOE) ɛ4 allele, have been implicated in sporadic AD (sAD), which represents the vast majority of cases. The APOE ɛ4 allele operates as a major genetic risk factor in sAD and modifies the age of onset [9–12]. The common molecular mechanism of the mutations or polymorphism of these genes associated with the pathogenesis of AD is their promoting effect of amyloid β (Aβ) generation due to an imbalance of APP metabolism [13] (Fig. 1).

## Pathogenesis of AD

AD is a multifactorial and heterogenous disorder, the pathogenesis of which is not completely understood. A currently most popular hypothesis on its etio-pathogenesis is the ‘amyloid cascade hypothesis', according to which an imbalance between the production and clearance of Aβ in the brain is the initiating event, ultimately leading to neuronal/synaptic degeneration and dementia [14]. Altered cellular processing of the amyloid precursor protein (APP) with increased production or a reduced removal of cleavage products such as Aβ is a major factor for amyloid deposition [15]. APP is a member of a family of integral membrane proteins, which has been identified by sequentiation of Aβ deposited in AD brains [16]. On chromosome 21, 19 alternatively composed exons are coded for APP, which is up to 770 amino acids long. It is ubiquitous and expressed in various isoforms, possesses a long, extracellular/intravesicular N-terminal and a short cytoplasmic C-terminal end. All isoforms contain the full Aβ sequence. APP over-expression is the consequence of different stimuli, such as ischemia, trauma or inflammation *in vitro* or is achieved in transgenic (tg) animal models [17].

Consistent with this hypothesis, both intracerebral infusion of Aβ in FTDP-17 tau mutation P301L-expressing tg mice and crossing these animals with APP tg 257G mutations, showed exacerbation of neurofibrillary pathology [18, 19], and in the 3 **×** tg-AD mice, Aβ deposition was found to precede neurofibrillary pathology, being more severe than in double tg 2× tg-AD mice [20, 21]. However, in these mice harbouring PSEN1 APP and tau (P301L) synaptic dysfunction manifested in an age-related manner before plaque and tangle pathology [21], suggesting that plaque and tangle pathologies contribute to cognitive dysfunction at later points in time.

**Fig. 2:**
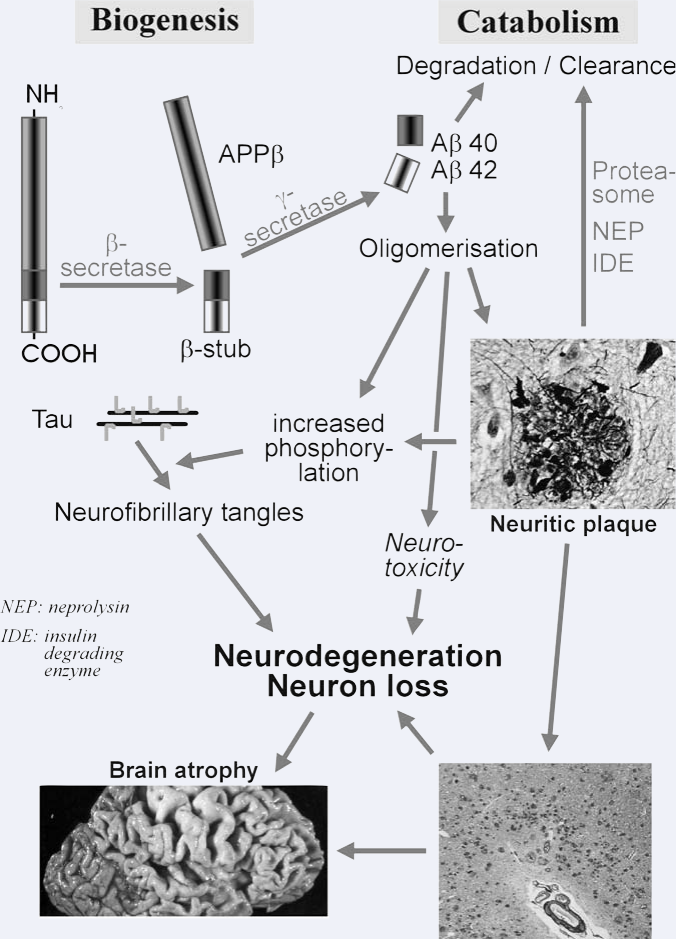
Molecular pathogenesis of Alzheimer disease. Deposition of amyloid β peptide after cleavage of amyloid precursor protein (APP) and hyperphosphorylation of tau protein induces formation of plaques, neurofibrillary tangles and neurodegeneration with neuronal and synapse loss.

But, up to the present, no data from human conditions do support the amyloid cascade hypothesis (see [22]), and in the brains of AD patients there are no definite proofs for overexpression of the large type I transmembrane protein APP. Aβ is produced from APP as a result of two sequential proteolytic cleavages involving a membrane-bound aspartyl protease (β-secretase, BACE1) and two homologous membrane proteases (PSEN1 and PSEN2), corresponding to λ-secretase activites [23]. Inside raft clusters, APP is suggested to be cleaved by β-secretase and outside rafts by α-secretase [24]. This enzyme cleaves APP in the middle of the Aβ region to generate a secreted extodomain (α-APP) and a shorter α-cleaved COOH-terminal stub of APP (α-CTF) that is also cleaved by 7-secretase. Aβ-42, also suggested to be neurotoxic, is produced by proteolytic cleavage – numerous proteases are capable to cleave Aβ at multiple sites *in vitro*[25]– and accumulates as fibrillary aggregates in plaques and vessel walls (Fig. 2). The car-boxy-terminal fragments (CFTs), which show important neuropro-tective and neurotrophic activities, are transported by anterograde axonal transport and have functional importance for synapse formation [26]. Latest progress in understanding the role of soluble Aβ oligomers in AD has been reviewed recently [27].

The isolated role of Aβ in the pathogenesis of AD has been examined *in vivo* without inhibition of the complex cascade in the processing of APP tg mice carrying the construct of murine Aβ under control of the NF-L promoter showed significant pathological changes with intraneuronal expression of Aβ, widespread apoptosis and reactive gliosis finally leading to death. When Aβ peptide is transported by binding to an NCAM-signal peptide into the extracellular spaces, no pathological changes occur [21]. These data and the recent demonstration of intraneuronal accumulation of Aβ-42 as a probable product of normal neuronal metabolism with damage to synapses already before Aβ deposition in the brain tissue suggest its important role in the disease progression [17, 28] and as a source for extraneuronal aggregation of Aβ. The death of neurons, a prominent feature of AD, has been associated with the release of oligomeric intracellular Aβ-42 into the surrounding tissue, which may stimulate the production of amyloidogenic fragments of APP, amplify the levels of intracellular Aβ in neighbouring cells, and act as a nidus for the deposition of secreted Aβ[29, 30]. Recent studies, however, indicate that intraneuronal Aβ immunoreactivity, observed already in young human control brains, is not a predictor of brain amyloidosis or neurofibrillary degeneration [31]. There are only weak correlations between amyloid deposits (numbers of plaques) and neuronal dysfunction in both human brains and tg mice [32], and the rate of brain atrophy is not determined by the amount of insoluble Aβ in the grey matter [33]. However, there is a direct relationship between synaptic loss and soluble Aβ which has induced the Aβ-derived diffuse diffusible ligands (ADDL) hypothesis that suggests that soluble oligomeric are highly neurotoxic proteins and might be the culprits damaging function and plasticity of synapses, thus disrupting cognitive functions [27, 34–37]. Exogenous, soluble (blood-borne) Aβ peptides can enter the brain through a defective blood-brain barrier and bind selectively to neurons [38].

APP tg B6SJL mice immune hyporesponsiveness to Aβ suggesting that an Aβ-specific impaired adaptive immune response may contribute to neuropathology [39]; increase of autoantibodies to Aβ in AD patients suggests that a humoral immune response to Aβ may promote neuronal degeneration, a process with implications for future vaccine-based therapies of AD [40]. Short Aβ immunogens have been shown to reduce cerebral Aβ load and learning deficits in an AD mouse model in the absence of an Aβ-specific cellular immune response [41], and it is anticipated that selected Aβ human monoclonal antibodies could reduce and inhibit Aβ deposits in brain [42] in the absence of Aβ-specific T-cell reactivity [43], which requires careful antigen and antibody selection to maximize efficacy and minimize adverse events [44].

Almost in parallel with the deposition of Aβ in plaques, tangles have been shown to be composed of abnormally phosphorylated tau protein [45]. Tau is a normal axonal protein that binds microtubules, promoting their assembly and stability, its hyperphosphorylation causing disassembly of microtubules and thus impaired axonal transport, compromising neuronal and synaptic function [46, 47]. Tau protein forms abnormal fibrils in AD and other neurodegenerative diseases, called tauopathies. It is highly soluble and adopts a natively unfolded structure in solution, whereas in its phosphorylated, filamentous form, *e.g.* neurofibrillary tangels (NFT), small segments of tau adopt a β-conformation, and interact with other tau moledules, giving rise to a fuzzy coat of the filaments [48]. The relationship between Aβ and tau pathologies and their impact on the pathogenesis of AD are still a matter of controversy. Biochemical studies on the course and distribution pattern of their pathologies suggest independent but synergistic affects [49]. Some authors suggest that AD is a tauopathy fueled by APP dysfunction [50], and that tangles either antecede plaques or – less likely – are independently found [51]. Others believe that increased formation and deposition of Aβ caused by dysmetabolism of APP that normally occurs in neurons and synapses [52, 53] induce early pathological phosphorylation of tau *via* activation of caspases, the key enzymes in the final cascade of programmed cell death, inducing the formation of NFT [54]. Activity of caspase-6 that cleaves tau at amino acids 402 and 13 was detected in mild to severe AD cases [55], particularly in the entorhinal cortex in absence of plaques and tangles, indicating that it is activated as an initial site affected in AD, resulting in neurodegen-eration and inflammation. Recent developments suggest that axonal transport of APP influences Aβ-deposition and APOE ɛ4 influences both axonal tau phosphorylation and amyloid-induced pathology, thus connecting the pathogenic mechanisms of Aβ, tau and APOE ɛ[56]. An ‘alternative’ amyloid cascade hypothesis suggests that amyloid deposition does not represent the initial cause of AD, but is the consequence of other processes, such as abnormalities in proteins regulating the cell cycle, oxidative stress, mitochondrial dysfunction with disruption in neuronal energy metabolism [57–60], and may represent a neuroprotective reaction [61, 62]. Another, alternative interpretation of the amyloid hypothesis speculated that accumulations of intramembranous Aβ peptides might affect the functions of APP itself and the assembly of PSEN 1, nicastrin and other complexes [63], while another new hypothesis about Aβ toxicity gaines some support regarding its effects on ion channel functions [64]. However, due to deviating findings in tg mouse models of AD [17, 65], currently neither the exact course of the molecular cascade leading to neurodegeneration in AD (Fig. 2) nor the final pathological cascade leading to neuronal death is fully recognized. The different etiologic factors suggested to be active in the development of AD pathology may lead to some common downstream pathogenic events that ultimately precede the disease, but further extensive basic research, including the use of animal models, is warranted [66].

### Alzheimer pathology and diagnosis

Sporadic AD is a slowly progressive disorder with insidious onset and duration of around 10–12 years. Its diagnosis and the differentiation from other dementing disorders is difficult and only in rather progressed stages of the disease, when memory and other cognitive disorders are severe enough to have a significant impact on the everyday life of the patient, the diagnostic accuracy when using all currently available tests is around 90%. Prior to a pathological diagnosis, only a ‘possible’ or ‘probable’ diagnosis of the disease can be made using current criteria [67–70]. A definite diagnosis of AD is only possible by the histopathological detection of its characteristic changes, the accumulation of extra-cellularly aggregated Aβ in plaques and cerebral vasculature (cerebral amyloid angiopathy / CAA) and intracellular and neurit-ic deposition of hyperphosphorylated tau protein (NFTs, neuropil threads and neuritic plaques), associated with loss of synapses and neurons in specific brain regions and loss of neuronal connectivity as a major cause of dementia (see [71, 72]). Although most of these changes are non-specific, they represent the major morphologic markers of AD, the (semi)quantitative assessment of which are the basis of current morphological criteria for the diagnosis of AD: CERAD [73], Braak & Braak, and National Institute on Aging – Reagan Institute (NIA-RI) criteria combining both CERAD and Braak stages [74], which gave good correlations between clinical data and pathological findings between AD and non-demented controls [72]. The degree of dementia usually correlates significantly with the density and extension of tau pathology, while it shows only weak correlations with the number of senile plaques but better ones to the total load of soluble Aβ (see [72, 75]).

Autopsy studies indicate that tangle-tau pathology first appears in the medio-basal temporal lobe, particularly in the (trans)entorhinal cortex, progressing *via* the hippocampus to the isocortical association areas with final involvement of subcortical nuclei [76, 77]. This distribution pattern correlates with early impairment of memory due to interruption of the GABAergic ‘perforant path', which connects the hippocampus with other areas of the brain, thus isolating the hippocampus from ingoing and outgoing stimuli. The rather stereotypic development of tau pathology differs from the phases of amyloid deposition usually beginning in the neocortex with anterograde involvement of the allocortex into regions that accept neuronal projections of already involved areas [78].

Neurodegeneration in AD is estimated to start 20–30 years before clinical onset, during which preclinical phase plaque and tangle load increase and at a certain threshold the first symptoms appear [1, 72]. This clinical phase is usually designated ‘mild cognitive impairment’ (MCI), during which subjects have measurable cognitive deficits, but which are not sufficient to fulfill criteria for any specific dementing disease [79, 80]. It has been suggested to constitute a transitional stage between normal aging and AD, but data show that many patients with amnestic MCI have early neu-ropathological changes of AD including hippocampal synaptic loss and, thus, represent early AD [81–83], but many cases with CDR 0.5 show variable pathologies not restricted to AD [84–86]. While some of these patients may remain stable or even may improve, many of them convert to AD with an annual conversion rate to clinical dementia of 10–19%[87, 88]. Preclinical AD usually shows more severe neuritic pathology than ‘normal’ aging with variable intensities of tau and amyloid biochemistry. The combination of clinical, neuropsychological and imaging methods differentiated various subtypes of MCI [89–92], which, however, did not show significant differences in their annual conversion rate to dementia [88].

### Biomarkers of AD

Biomarkers are required to improve the diagnostic sensitivity and specificity and to monitor the biological activity of AD in terms of the burden of neuronal involvement and the tempo of disease progression. In view of the advancing scientific knowledge regarding biomarkers for AD/MCI, it was proposed to incorporate those biomarkers in revised diagnostic criteria in the future [91–93]. Biomarkers will initially supplement our more traditional neuropsychological and imaging markers and may progress to useful surrogate clues to the pharmacological action of anti-dementia compounds [94–100].

### CSF biomarkers

Senile plaques in AD brain are mainly composed of Aβ-42, while CAA mainly contains Aβ-40 [101, 102]. An inverse relation between *in vivo* amyloid load and CSF levels of Aβ-42 has been found in human beings [103]. CSF of AD patients even in early phases of the disease shows decreased values of Aβ-42 and, together with increased total tau (tTau) or phosphorylated tau (pTau), suggests the presence of cognitive dysfunctions even in healthy elderly subjects [104, 105]; CSF tau/Aβ-42 ratio is a prediction of cognitive decline in non-demented older adults [106–110] and with MCI [110–113]. Together with medial temporal lobe atrophy on MRI [114–118] or FDG-PET [119] and presence of the APOE ɛ4 allele, these markers predict dementia in mild cognitive impairment or higher risk for developing AD [83, 89, 120] as does rCBF-SPECT [121] and cerebral hypoperfusion induced by fMRI [122] and plasma Aβ-42, medial temporal lobe atrophy and homocysteine [123]. *Antemortem* CSF levels of Aβ-42, tTau and pTau-Thr231 have been reported to reflect the histopathological changes observed *postmortem* in the brains of AD cases [124, 125]. The CSF levels of tau are markedly increased in patients with diffuse axonal injury in head trauma, which revert on clinical improvement [126]. Thus, bulk of the evidence supports that CSF reflects the state of the brain protein metabolism; CSF levels of toxic-advanced glycation end products (TAGE) may also help in early detection of AD [127]. At present, the combination of elevated CSF tTau or pTau proteins and low CSF Aβ-42 are the only biomarkers with enough sensitivity and specificity to serve as useful diagnostic biomarkers capable of distinguishing AD from other dementias in the early stages [96, 128–136], PD patients with dementia (PDD) from those without [137] and DLB [138], while CSF serpin levels did not improve the diagnostic classification of AD *versus* DLB [139]. They are possible markers for severity and abundance of symptoms in AD [71, 105, 140–144] (Table 1). Other studies demonstrated that significant elevation of BACE 1 levels and activity in CSF is an indicator of MCI or other neuroimmune markers indicating AD [145, 146]. Whereas progressive increase of tTau CSF concentrations was found from early to advanced stages of AD [147], soluble Aβ-42 has been shown not to be related to the degree of cognitive impairment [140], and Aβ-42/40 ratio but not Aβ-42 alone correlates with pTau in patients with low and high CSF Aβ-40 load [148]. Recent longitudinal studies showed that levels of CSF Aβ-42 and tTau, but not pTau at threonine 181, increased over time in a memory clinic patient cohort with comparable changes in all diagnostic groups. However, the cross-sectional difference between diagnostic groups exceeded by far the longitudinal changes within individuals, suggesting that these biomarkers are not sensitive as markers of disease progression [149]. The causal relations between progress of Aβ load and plaque density in the brain and CSF Aβ reduction are not completely understood, but they were attributed to depletion of the monomeric protein into oligomeric soluble and insoluble forms in the brain and increased Aβ deposition in plaques [94]. Age and APOE ɛ4 allele accelerating pathogenic Aβ-42 brain deposition starting in late middle age in persons with normal cognition are causing decrease of CSF Aβ-42 but not the Aβ-40 concentration [150], while CSF Aβ-40 levels in frontotemporal dementia (FTD) are decreased [151] and fluctuations of CSF Aβ levels have been found in individual subjects [152]. Levels of ApoE in CSF are correlated with tau and 24S-hydroxycholesterol in patients with cognitive disorders [153, 154]. Extreme CSF Aβ levels identify familial eoAD and loAD PSEN1 mutations and, thus, can be useful endophenotypes for genetic AD [155] as well as for children with Down syndrome [156], but inverse relations between *in vivo* amyloid imaging load in human brain and CSF Aβ-42 have been found [103].

**Table 1:** Well-documented CSF markers in dementias [69, 103, 142]

Test	Cutoff (pg/ml)	Specificity / Sensitivity	MCI →AD
		clin. AD *versus* controls /*versus* non-AD dementias	
tTau	300–500	0.85–1.0 / 0.40–0.89	SP 0.70
	250	AD *versus* LBD SP 0.76	
	2130	CJD *versus* AD 1.0 / 0.93	
Aβ-42	375–500	0.47–1.0 / 0.78–1.0 // 0.6–0.9 / 0.85	1.0 / 0.83
Aβ-40		Not relevant (no differences)	
Aβ ratio (40 / 42)		0.82 / 0.58	
Tau /Aβ ratio	0.5	0.82 / 0.58	AD *versus* LBD 0.84 / 0.79
Tau × Aβ ratio		0.88 / 0.69	
tTau + Aβ-42		0.81–1.0 / 0.63–0.89; 0.75–0.93 / 0.71	0.90 / 0.83
		AD *versus* LBD SP 0.67;	
		AD *versus* VaD 0.58–0.83 / 0.90	
		AD *versus* FTD SP 0.58	
pTau		0.80–0.85 / 0.90–0.94	MCI →AD 1.0 / 0.65
		AD *versus* FTD 0.92 / 0.90	
pTau + Aβ-42		0.97 / 0.89	0.80 / 0.73–0.89
		AD *versus* FTD 0.93 / 0.72	

Formulas:

Specificity (SP) = definitely negative / (definitely negative + wrong positive)

Sensitivity (ST) = definitely positive / (definitely positive + wrong negative)

The aspects of Aβ as a biomarker for AD have been reviewed recently [25], as well as the variable patterns of CSF Aβ between synucleinopathies and tauopathies [157] or other neurodegenerative dementias [158, 159]. While initial studies suggested that CSF pTau protein correlates with neocortical neurofibrillary pathology in severely demented AD patients and may serve as *in vivo* surrogate marker of tangle tau pathology in AD [125], other recent studies showed no association of CSF biomarkers (Aβ-42, t- and pTau) with APOE ɛ4, plaque and tangle burden in autopsy-confirmed AD [160, 161]. Increased CSF tau is also seen in other CNS disorders with neuronal loss (degenerative, inflammatory, vascular, tumours), with highest CSF tau concentrations in Creutzfeldt-Jakob disease (CJD) and brain infarcts, the former showing a dissociation between tTau and pTau [162], while AD and AD+CVD display a similar neurochemical phenotype with increased tau and diminished Aβ-42, with some overlap between AD and VaD, the latter showing a decrease of CSF Aβ-38/40 [158, 163], as well as in plasma [164]. CSF A**β** peptides have also been suggested to distinguish between DLB, PDD, AD and FTD [158, 165]. In relatively young dementia patients, CSF neurofilament protein levels may play a role in the discrimination between FTD and early onset AD, especially in combination with Aβ-42 and pTau analysis [166]. CSF antimicroglial antibodies are also a putative marker of an ongoing inflammatory process in AD [167]. However, since most studies evaluating biomarkers are compared only with clinical rather than pathological diagnoses, the concept of *in vivo* surrogate markers should be further explored. Re-evaluation using immunoprecipitated CSF samples of neuropathologically defined dementing disorders will be necessary to determine, whether Aβ-40 and Aβ-42 will be applicable as neurochemical dementia markers. Comparative proteomics of CSF detecting a large number of hitherto unknown proteins in autopsy-confirmed AD and non-demented elderly subjects may provide a further means to diagnose and assess AD with 90% sensitivity and 83% specificity [129, 168–175] and to distinguish between various degenerative dementias [176]. Preliminary studies showed that redox-reactive antibodies in CSF may represent valuable biomarkers for AD diagnosis [177].

### Plasma biomarkers

Plasma total amyloid or Aβ-42 is increased in cases of familial AD and trisomy 21 [178, 179], but were not consistently related to diagnosis in clinic-based cross-sectional studies of typical late-onset AD [178, 180–185].

Elevated levels of Aβ-42, low levels of Aβ-40 and a reduced Aβ-42/Aβ-40 ratio in plasma of old subjects indicate a conversion of cognitive normality to MCI or AD [186–188], and have been found in geriatric depression [189]. Others, however, found that a reduction in Aβ-42 plasma levels may be a marker for AD status, specifically a transition from normal status or MCI to AD [190], rather than a marker for neurodegenerative processes occurring in the disease [191]. Due to a high intra- and inter-personal variability of serum and plasma Aβ levels [192], plasma levels of Aβ-40 and Aβ-42 are not robust correlates of histologically or biochemically assessed amyloid burdens in brain and the origin of plasma Aβ is not completely understood [193]. Plasma Aβ levels are probably modulated by peripheral and brain metabolism and clearance as well as by transport across brain, CSF and vascular compartments. Although all forms of brain Aβ are elevated in AD, the weak correlations of the various brain Aβ measures in AD suggest that they may reflect distinct biochemical and morphological pools of Aβ[194]. The normal equilibrium between CSF and plasma Aβ may be disrupted with the initiation of Aβ deposition in the brain [195], but plasma and CSF Aβ levels are not correlated in AD [196], and Aβ-42 plasma levels are considerably influenced by concomitant medication [197], in particular by insulin treatment [198]. It is not unexpected, therefore, that plasma and brain Aβ levels are not strongly correlated. Reduction of CSF and plasma Aβ has been observed in cerebral amyloid angiopathy (CAA), amyotrophic lateral sclerosis, and dementia with Lewy bodies (DLB), and in CJD a dissociation of the Aβ peptides in CSF has been observed [199]. Measurement of CSF tau together with serum heart type fatty acid-binding protein (CH-FARP) to CSF tau may represent marker candidates for the differentiation between AD and DLB [200]. It is generally agreed that plasma Aβ-42 levels alone seem not to be reliable biomarkers for MCI and AD [123, 183, 201], while a decrease in the ratio of plasma Aβ-38/-40 peptides is considered a blood-based biomarker for vascular dementia, its diagnostic accuracy resembling that of CSF markers for AD [164]. Furthermore, blood inflammatory markers, like CRP, interleukin-6, etc., are markers for VaD [164, 202] or are increased before clinical onset of both AD and VaD [203], while other inflammatory molecules are associated with AD progression [204]. If plasma Aβ is a risk factor for AD as is suggested by several longitudinal cohort studies, the relevant Aβ levels may be those seen 5–20 years before death [187, 205]. Furthermore, changes in plasma Aβ-42 may be a biochemical predictor of rivastigmine treatment efficacy [206]. Proteomic analysis of plasma revealed higher concentrations of α-1-antitrypsin and apolipoprotein J in AD patients [207]. Recent proteomic discovery of various plasma signaling proteins may allow the development of a simple, cost-effective test for AD [208, 209].

Plasma homocysteine has been shown to be directly related to Aβ-40 levels, while the association with Aβ-42 was not significant, suggesting that homocysteine is related to aging but not specifically to AD, but it could interact to affect AD risk and cognition in PD [210, 211]. Recently, some proteome-based plasma serum biomarkers were shown to be specific for AD and to correlate with disease severity, although alternative assays will be necessary to improve sensitivity and specificity [212, 213].

### Platelet biomarkers

Among changes of blood platelets, APP abnormalities have been suggested to predict conversion of MCI to dementia of the AD type [214, 215], and have been correlated with membrane fluidity and cognitive decline [216]. Increased BACE-1 activity in AD [217] and increase of monoamine oxidase B expression in blood platelets – and in brain – of demented patients with both AD and PD has been repeatedly confirmed [218–220]. Recently, increased platelet phospholipase A2 activity has been detected in patients with AD, VaD and ischemic stroke [221].

### Structural biomarkers

The value of structural MRI-derived biomarkers for AD has recently reviewed [222, 223]. Medial temporal lobe atrophy (MTA) on MRI is sensitive to primary degenerative hippocampal atrophy in old subjects, but not specific for AD pathology, mild MTA score not being frequently associated with dementia [224], but for MCI [225]. The level of elevated PET amyloid ligand (11C)PIB uptake in patients with MCI / mild AD is suggestive of early AD process [226–229], even in non-demented individuals [230]. While analysis of CBF-SPECT, CMRgl-PET (glucose metabolism), proton spectroscopy (H-1 MRS), high-field strength functional MRI, voxel-based morphometry, increased activation of the mediobasal temporal lobe detected by fMRI, (R)-[(11)C]PK11195 PET to detect microglia, and other functional neuroimaging methods, in particular, combined PiB imaging and structural MRI, are used as sensitive markers for conversion of MCI into early AD [121, 231–241]. The same is true for accelerated rates of hippocampal atrophy and ApoE ɛ4 [114], FDG-PET [242], detection of subcortical hyperintensities [243–245] and MRI patterns of grey matter atrophy [246]. However, comparatively normal glucose metabolism in the presence of high frontal amyloid load suggests that amyloid plaque formation may not be directly responsible for neu-ronal dysfunction in this disorder [247].

A recent review of the neuropathological basis of MR-defined cerebral lesions indicated that the presence of lacunes and WMLs provide a good signal for VaD, whereas cortical and hippocampal atrophy in aging and dementia are complex with several processes converging on similar brain structures that mediate cognitive decline [248].

## ALZheimer ASsociated protein (ALZAS)

**Fig. 3:**
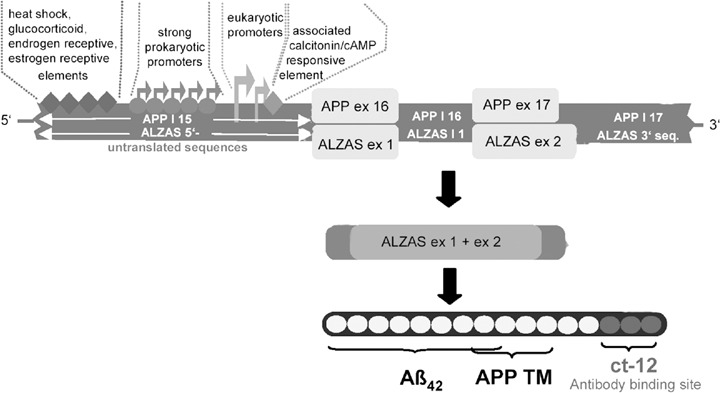
Scheme of the ALZAS gene structure (A), mRNA (B) and protein in the region of the APP gene (C) on chromosome 21. APP TM = transmembrane region.

Recently, a novel, hitherto unknown Aβ protein expressed in elderly patients with the diagnosis of probable AD was discovered on chromosome 21 within the APP region. This protein, ALZAS (ALZheimer ASsociated protein), with a 79 amino acid sequence contains the Aβ-42 fragment, the AP transmembrane signal and a unique 12 amino acid c-terminal, which is not present in any known allele of the APP [249–252]. In contrast to theoretic splice variants, it has its starting codon within the exon 16 and its coding sequence ends in intron 17 (Fig. 3). Reverse transcription PCR revealed the expression of the transcript of this protein in cortical and hippocampal brain regions as well as in lymphocytes of AD patients [249–251, 253, 254]. Using cloning methods, the genetic structure of ALZAS has been clarified [255].

**Fig. 4:**
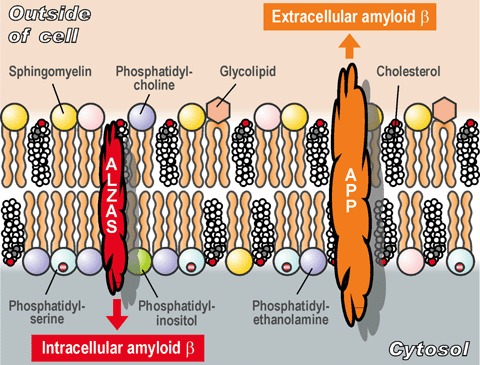
Hypothetical competing action of ALZAS with transmembrane APP.

Regulating the access of α- and β-secretases to APP and ALZAS seems to be of importance in view of recent CSF studies providing a direct evidence that Aβ-fragments (truncated Aβ 1–16, 1–33, 1–39 and 1–42) in CSF distinguish sporadic AD from non-demented controls with an overall accuracy of 86%[141]. The finding that Aβ 1–16 was the most abundant peptide may indicate that ALZAS with its predicted β-helical structure may be a substrate for α-secretase [171, 253]. Moreover, it could act as molecular chaperone that binds APP and assists in altering its conformation. ALZAS, as a specific Aβ protein, may compete or even out-compete APP for the APP transmembrane transit site in specific neurons, paralleled by intraneuronal neurotoxic accumulation of Aβ[250] and activating phospholipases, resulting in an ongoing membrane decay and synaptic disruption in AD (Fig. 4) [256]. Currently, little is known about the process in which such peptides, like Aβ, ALZAS or prions, may induce changes in neuronal phenotypes and microglial activation [257].

**Fig. 5:**
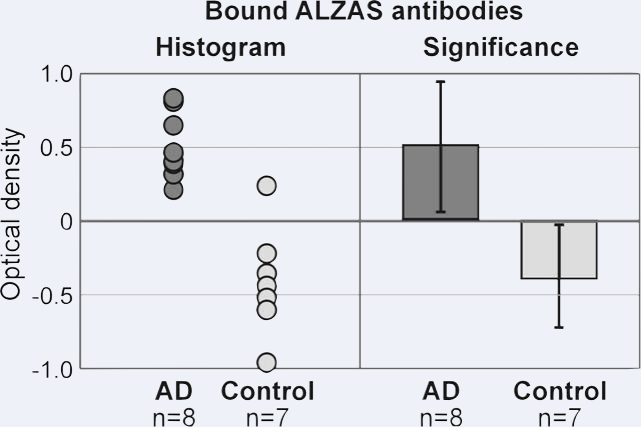
ELISA studies of EDTA plasma samples (dilution 1:20, preincubation 4 hrs, urea 1 M at 37°C). Detection of ALZAS IgG antibodies in eight AD patients and seven controls (signal: optic density /OD/ 492–620 nm).

Pilot studies in serum of patients with probable AD have detected an up to tenfold increase of the ALZAS antibody titre directed against the non-amyloidotic C-terminal of this protein. Serum ELISA studies revealed the highest titres in early stages of the disease, *i.e.* in patients with presymptomatic AD or MCI, but moderately increased titres in fully developed and autopsy-confirmed AD. Low or undetectable anti-ct 12 titres were found in healthy age-matched subjects or young controls (Fig. 5). Maximal values were found in the sera of patients over age 65 years who had been diagnosed as ‘depressed’ without recognizible cognition disorders. It should be emphasized that depression is common in early stages of AD (up to 87%) and is associated with significant morbidity [258–261]. Increased hippocampal tangles and plaques in patients with AD and a lifetime history of major depression suggest an interaction between both disorders [262].

### ALZAS immunohistochemistry

**Fig. 6:**
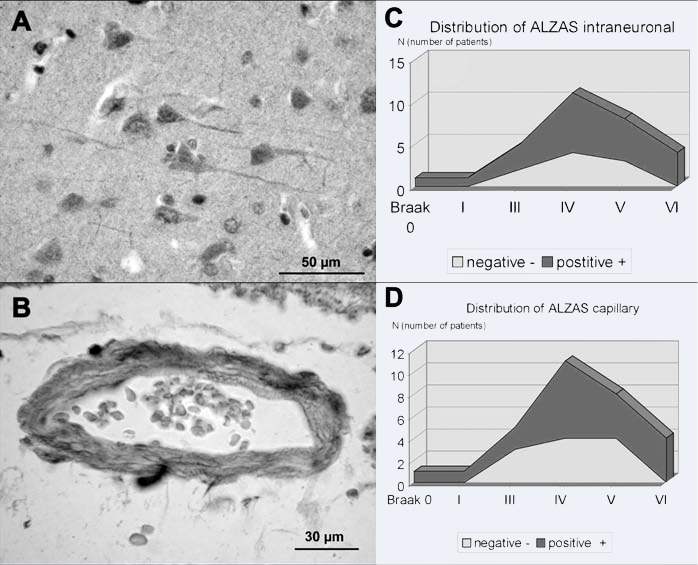
Immunostaining of ALZAS protein in human brain tissue showing intraneuronal deposits in frontal cortex (**A**) and capillary immunostaining (**B**). Distribution of ALZAS intraneuronal and intracapillary staining in frontal cortex of AD patients significantly correlates with neuritic Braak stage (**C**, **D**).

Intraneuronal deposition of ALZAS protein has been demonstrated in brain slices of frontal cortex and hippocampus from autopsy-proven AD patients, using a chicken antibody, cleaned by high affinity chromatography, whereas controls were negative (Fig. 6). Wilcoxon statistics of the intraneuronal and capillary immunostain-ing of ALZAS showed significant correlations with Braak stages and NIA-Reagan classification of AD (Fig. 6A and B). Nine cases (30%) were ALZAS negative, one (3.3%) showed positive ALZAS neuronal but negative capillary decoration, while positive neuronal and capillay decoration was present in 20 cases (66.7%). In *postmortem* AD brain, ALZAS immunoreactivity was located intracellularly in neurons and in the inner vascular membranes (Fig. 6B and C).

### ALZAS-mRNA studies

To further verify the postulated ALZAS protein, the ALZAS-mRNA isolated from human *postmortem* AD brain was amplified and sequenced using primers from regions of ALZAS gene from frontal cortex of AD brains and age-matched controls. ALZAS mRNA expression was examined by qRT-PCR. The received sequence correlated with the postulated mRNS sequence of the APP-exons 16 and 17 as well as to parts of the adjacent introns APP I 15 and 17 [253].

**Fig. 7:**
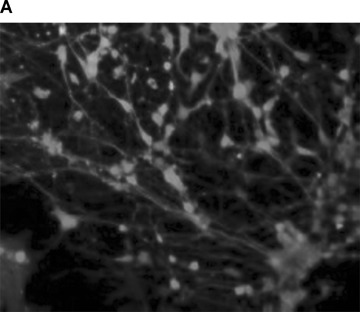

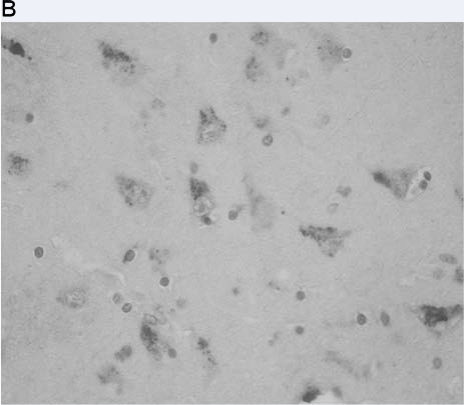
ALZAS protein is expressed in transgenic mouse stem cells (**A**) and intraneuronally in frontal cortex of human AD patient (**B**). Aβ-specific 4G8 immunostaining.

An initial quantification of ALZAS mRNA in different tissues using RT-PCR showed that ALZAs is transcribed in all examined tissues (lymphocytes, cerebral cortex), but a preliminary assessment of the results did not show essential differences in the transcription rates between AD patients and healthy controls. Moreover, cell culture studies, using mouse embryonic stem cells that harboured an ALZAS transgene, which included the calcitonin responsive promoter, showed that ALZAS expression was initiated by adding calcitonin to the culture. In cultured transgenic mouse stem cells, ALZAS was located in neuronal plasma membranes of differentiating embryonic ES cells. Intraneuronal immunolabling of cortical sections from AD brain with Aβ specific 4G8 antibody showed similar decoration of neurons as in transgenic mouse stem cells (Fig. 7). Moreover, the ALZAS gene was cloned in an inducable expression vector for further transfection in human neu-roblastoma cells (SH-SY5Y) [253].

Despite increased ALZAS antibody titres in AD patients, the transcription in the examined tissues of AD patients and controls appears similar, and there are only some differences between tissues, with highest transcription and lowest transcription differences between ALZAS and APP in the blood. Up to now, the role of ALZAS in cell metabolism is highly speculative. Further studies should clarify whether ALZAS *per se* or in interaction with his ‘bigger brother’ Aβ-42 leads to the known disorders of metabolism and protein aggregation in AD brain. It is further unknown which type and severity of ALZAS expression is necessary, which cells are predominantly involved and by which mechanisms the pathogenic expression of ALZAS in AD patients is induced. Cloning of the ALZAS promoter region in an expression vector may enable us to find transcription factors that selectively influence the gene expression and thus probably may trigger the pathogenesis of AD. Individual findings in hitherto examined blood probes and comparison of sequences of the promoter region could indicate probable hormonal reactions.

### ALZAS as a future biomarker?

**Fig. 8:**
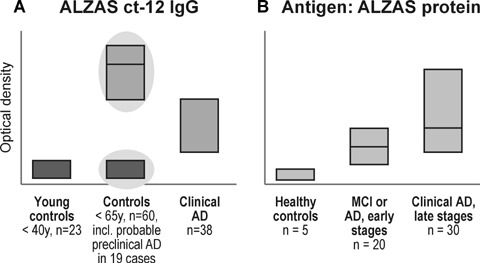
ELISA studies of EDTA plasma samples to detect anti-ALZAS IgG revealed increased levels in MCI patients (*n*= 19) and, less severe, in patients with AD dementia (*n*= 38) as compared to young and aged controls (**A**). There is only mild increase of ALZAS protein in plasma of MCI patients but more severe in AD dementia (**B**).

The plasma of AD patients in comparison to age-matched healthy controls contains increased concentrations of ALZAS antibodies, which could be confirmed by repeated studies [255]. Using an ALZAS-Capture enzyme-linked immunosorbent assay (ELISA), specific IgG antibodies were detected that exclusively are directed against the C-terminal end, coded at the intron-region of APP. Preliminary data also revealed an increase of ALZAS peptide, possibly acting as autoantigene, in the serum of later stages of AD (Fig. 8B). However, the distinction between AD and other dementing disorders, such as vascular dementias, has to be examined by further studies. Significant elevation of serum ALZAS IgG was detected in patients with early stages of dementia, in whom neuropsychological test results were still above the threshold levels of dementia. Even in patients with MCI who later developed AD dementia, at base level increased ALZAS values were detected. This might be interesting for an early diagnosis of AD and could be a valuable sign for early treatment. However, there is considerable variability of the specificity and sensitivity of ALZAS protein depending on the compared groups, the statistical methods and the cut-off values [253, 254]. In addition, the influence of age has to be considered, which is currently under investigation.

## Conclusions

Progress in our understanding of the molecular pathology and pathogenesis of AD and its relationship to brain aging have provided clinically meaningful advances in the development of biomark-ers that are based on biochemistry and neuroimaging methods. They should have the potential to provide effective measures of early diagnosis, biological acitivty, disease outcome, and of indicating surrogate endpoints for their clinical use in diagnostic and preclinical purposes and for monitoring treatment. Because most clinical criteria lack specificity, particularly early in the course of disease, and most studies related marker values with clinical criteria and only very rarely with neuropathological diagnoses, quoted sensitivities and specificities for putative biomarkers are difficult to interprete. The currently best validated CSF biomarkers, pTau and Aβ-42, with a reported sensitivity and specificity of around 90% for the diagnosis of AD, show at least some, if inconstant, correlations with cerebral tau and Aβ pathology [125, 130, 160, 161]. As biomarkers in plasma several substances have been examined, *e.g.* isopostane, 3-nitrotyrosin, α-1-antichymotrypsin, interleukines, C-reactive protein, C1q complementary systems, 24S-hydrocholesterol, homocysteine, but none of these markers had enough sensitivity or specificity to diagnose AD [100, 134]. The same appears to be true for serum Aβ peptides, which do not reflect the Aβ load in the brain. Although in combination with MRI studies of the mediobasal temporal lobe they may indicate conversion of cognitive normal seniors to MCI or AD [123], but alone they are not reliable biomarkers for AD. In the future, assessment of autoantibodies in serum, serum platelets and lymphocytes against Aβ and RAGE (receptor for advanced glycation end products) appear of increasing interest [122, 123, 263, 264], and may indicate close relations between AD and autoimmune disorders [265].

Chronic inflammation with microglia activation is believed to play a central role in the pathogenesis of lesions in the central nervous system of patients with AD or multiple sclerosis (MS). In AD, abundant microglia activation is present in the affected cortex [266]. Microglia cells activated *in vitro* under AD-like conditions, such as by the addition of Aβ peptide to the culture medium, produce a variety of pro-inflammatory and toxic cytokines and mediators [267–269]. Such toxic factors have been suggested to augment or promote amyloid deposition and neuronal degeneration [270–272]. In contrast, active immunization or passive transfer of specific antibodies against the Aβ peptide have been shown to reduce amyloid deposits in tg models of AD and in patients [273]. Microglia cells and astrocytes are believed to play an important role in the clearing of Aβ deposits [274–277]. This may occur in experi-mental models even in a bystander fashion without the involvement of specific adaptive immune responses against Aβ[41].

Although MS has long been considered a demyelinating disease of the white matter, extensive cortical demyelination has been shown as a characteristic hallmark of the pathology of progressive MS [278, 279]. At this stage, profound inflammatory infiltrates composed by T and B lymphocytes in the meninges are associated with severe microglia activation in the cortex and the formation of widespread subpial bandlike demyelinated lesions [280–282]. Microglia cells activated in the MS brain also produce pro-inflammatory cytokines and toxic factors, which contribute to demyelination and axonal lesions [283]. To what extent chronic inflammation in the cortex of MS patients influences the development of AD lesions is so far unresolved.

A recent study of autopsy cases of MS, AD and age-matched controls showed profound microglia activation in both MS and AD cortices with similar patterns. But microglia activation in MS cortex, in contrast to that in AD and controls, correlated with lymphocyte and plasma cell infiltration into the meninges. Old MS patients showed AD pathology with comparable incidence as in the course of normal aging, and the density of Aβ plaques and NFTs did not differ between demyelinated and non-demyelinated cortical areas. These data suggest that the non-specific activation of microglia in MS cortex, which has been described to decrease Aβ load in experimental animals sensitized with copolymer I or adjuvant alone [41], has little or no interference with the development of cortical AD pathology [284], and does not play a major role in Aβ clearance in human beings, unless specific Aβ antibodies are present [275].

The ct-12 amino acid sequence (c-terminal immune response-elicitating sequence) that recognizes endogenous ALZAS antibodies, may convert involved cells to targets of microglial cells in brain tissue that induce an immune cascade and neuroinflammation processes as side effects of AD. In this connection, binding of ALZAS to the RAGE receptor, acting as amplification and activation factor of microglia, is under discussion. RAGE recognizes substances that are formed during chronic and age-related disorders, including A**β**[264]. Most recent studies demonstrate that the conversion of MCI to AD dementia is associated with inflammatory processes [285]. Microglia activation in AD appears to be driven by innate immunity [286–288]. A CD14-dependent inflammatory response to A**β** oligomers provides the basis for a hypothesis of a structural mimicry between aggregated, highly hydrophobic amy-loidogenic proteins and biophysically similar pathogen-associated microglial patterns, contributing to chronic neuroinflammation and neurodegeneration [289] that is suggested to be a consequence of microglial dysregulation and overactivation [290]. ALZAS peptide represents an additional source of Aβ, an auto-antigen due to its unique amino acid sequence (ct-12) and is predicted as pathogen that may induce both inflammation and neurodegeneration. The contribution of age-related loss of microglial neuroprotective functions to AD pathogenesis [286, 291] remains a plausible option. ALZAS as inducable gene could be a ‘missing link’ in the search for the causes of AD (Table 2 -[271]). Since ALZAS peptide like APP contains the transmem-brane signal and, therefore, concurs with APP for the deposition into the cell membrane, it may have relationship to membrane dysfunction and disorders of axonal transport in AD. While the clinical and pathological effects of active immunotherapy of AD with synthetic Aβ-42 are well established in animal models [273, 292], few reports have been made about the neuropathological findings in patients who received immunotherapy with Aβ-42 [274, 293–299]. These findings include various degrees of reduction in amyloid plaque burden, but persistence of tau pathology and CAA. Lymphocytic infiltration, characteristic of meningoen-cephalitis [295, 296], was not observed in all cases [297, 300], while others showed minimal effects in A**β** plaque reduction but severe CAA with brain haemorrhages [301, 302].

**Table 2:** Suggested role of ALZAS in pathogenesis and possible treatment of Alzheimer disease

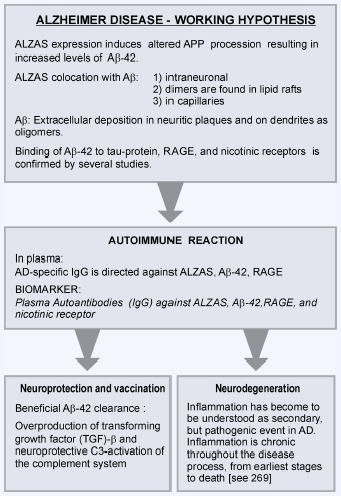

ALZAS protein, which has been detected in plasma in both early and late stages of AD, is suggested to represent an indicator of a dynamic equilibrium between both peripheral and brain degenerative changes, thus providing a reliable and simple diagnostic marker for AD by a simple, non-invasinve blood test. Further studies are warranted in order to validate the diagnostic sensibility and sensitivity of ALZAS ELISA serum tests in comparison to other currently used and future biomarkers in prospective clinico-pathological studies.
